# SpliceHarmonization: an integrated method for identifying RNA splicing events in therapeutics for splicing modulation

**DOI:** 10.1093/bioinformatics/btag111

**Published:** 2026-03-20

**Authors:** Yirui Chen, Haotian Zhang, Yu H Sun, Soumya Negi, Shaolong Cao, Zhengyu Ouyang, Baohong Zhang, Jessica Hurt, Dann Huh

**Affiliations:** Genomics and Computational Biology, Biomarkers and Systems Biology, Biogen Inc., Cambridge, MA 02142, United States; Genomics and Computational Biology, Biomarkers and Systems Biology, Biogen Inc., Cambridge, MA 02142, United States; Genomics and Computational Biology, Biomarkers and Systems Biology, Biogen Inc., Cambridge, MA 02142, United States; Genomics and Computational Biology, Biomarkers and Systems Biology, Biogen Inc., Cambridge, MA 02142, United States; Genomics and Computational Biology, Biomarkers and Systems Biology, Biogen Inc., Cambridge, MA 02142, United States; Data Science, BioInfoRx Inc., Madison, WI 53719, United States; Genomics and Computational Biology, Biomarkers and Systems Biology, Biogen Inc., Cambridge, MA 02142, United States; Genomics and Computational Biology, Biomarkers and Systems Biology, Biogen Inc., Cambridge, MA 02142, United States; Genomics and Computational Biology, Biomarkers and Systems Biology, Biogen Inc., Cambridge, MA 02142, United States

## Abstract

**Motivation:**

Splicing, a critical co-transcriptional process in eukaryotes, enhances transcriptome diversity by generating isoforms specific to cell types, tissues, or developmental stages. Recent advancements in splicing modulators have opened new avenues for targeting previously undruggable genes by inducing significant perturbations in splicing events. These developments underscore the need for comprehensive methods to accurately identify and compare splicing events. While several tools have been developed to detect local splice variants, inconsistencies across methods remain a significant challenge. To address this, we present SpliceHarmonization, an integrated approach that combines the strengths of rMATS, LeafCutter, and MAJIQ, enabling robust and reliable splicing analysis with event type annotations.

**Results:**

In a comprehensive evaluation using diverse simulated datasets, SpliceHarmonization streamlined and standardized the outputs from three detection methods into a unified format, thereby improving splicing detection with event type annotation and outperforming individual methods. By integrating the outputs from rMATS, LeafCutter, and MAJIQ, our approach not only enhanced identification of a wide range of splicing events but also effectively mitigated method-specific discrepancies. This integration led to an accuracy exceeding 0.8 and a recall of up to 0.5, with an observed increase in AUC of up to 10%. Furthermore, SpliceHarmonization demonstrated high sensitivity in detecting low-abundance and complex splicing events, providing annotations including genomic coordinates and event type.

**Availability and implementation:**

SpliceHarmonization is available at https://github.com/interactivereport/SpliceHarmonization

## 1 Introduction

Splicing is a fundamental co-transcriptional process in eukaryotic organisms, essential for gene regulation. This process diversifies the transcriptome by generating multiple mRNA isoforms from a single gene, tailored to specific cell types, tissues, or developmental stages. Recent advancements (Ratni *et al.* 2018, Darras *et al.* 2021, Keller *et al.* 2022) in modern drug discovery have led to the breakthrough of developing splicing modulators, serving as a new modality to treat various diseases. These modulators target splicing factors and the splicing process itself, thereby selectively altering the abundance of mRNA isoforms and offering therapeutic potential for diseases associated with splicing alterations. The ability to modulate splicing has also allowed researchers to explore targets previously deemed undruggable, providing a novel approach to influence gene expression at a co-transcriptional level (Schneider-Poetsch *et al.* 2021, Wheeler *et al.* 2024). In light of this therapeutic potential, it is critical to develop a robust method for monitoring and comparing both target and non-target activities across different molecules and treatment conditions throughout their development process.

While multiple computational methods have been designed with the purpose of detecting alternative splicing and quantifying changes between different conditions, significant challenges remain. Traditional approaches, like RNA-seq analysis tools, can identify splice variants but often suffer from inconsistent results across different methods and lack detailed event annotations. Tools such as rMATS (Shen *et al.* 2014, Wang *et al.* 2024), LeafCutter (Li *et al.* 2018), and MAJIQ (Vaquero-Garcia *et al.* 2016, 2023), each contribute uniquely to splice analysis. rMATS enables robust quantification of differential splicing events between experimental groups. LeafCutter identifies novel splicing variants by clustering intron-spanning reads without relying on transcript annotations. MAJIQ models local splicing variations by predicting and quantifying splice junction usage through a graph-based representation. Despite these capabilities, discrepancies in splicing event identification persist due to differences in algorithmic approaches and sensitivities to experimental conditions.

To fill these gaps, we developed SpliceHarmonization, an integrated method for splice analysis with detailed event annotations.

## 2 Materials and methods

### 2.1 Overall architecture

**Figure 1:**
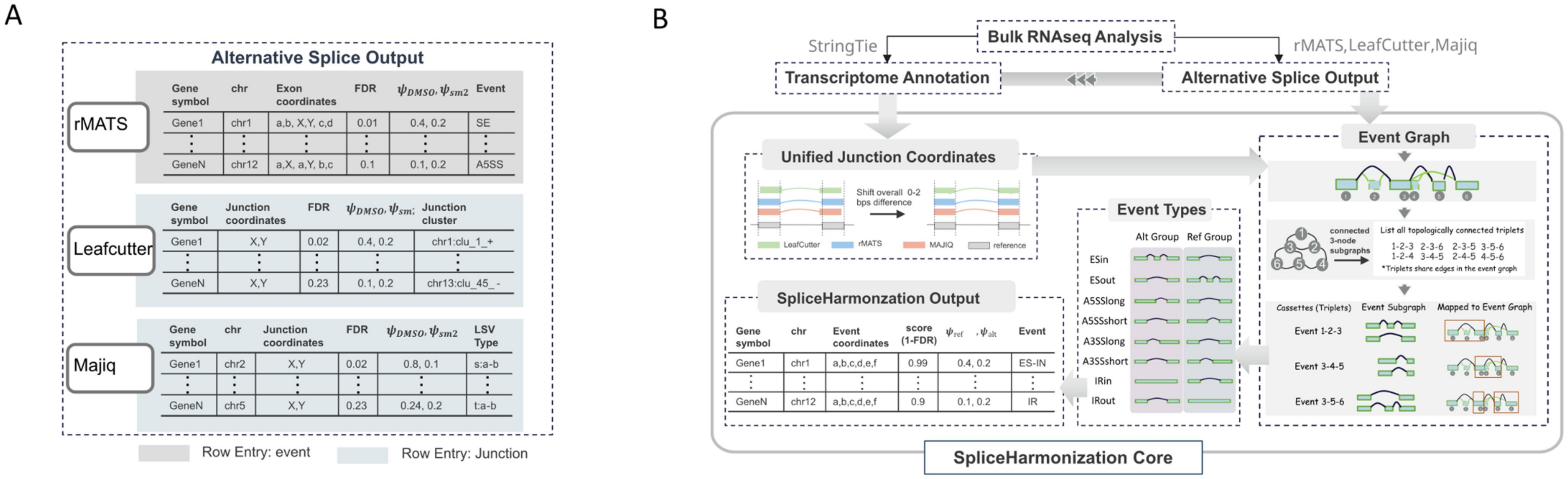
Overview of SpliceHarmonization. (A) Summarized outputs from three splice detection methods: rMATS, LeafCutter, and MAJIQ. Each row in rMATS describes a splice event, including FDR and Ψ values, while rows in LeafCutter and MAJIQ provide confidence score (1-FDR), Ψ and ΔΨ for each junction. (B) The SpliceHarmonization framework involves two key steps: (i) unifying junction coordinates across methods and (ii) establishing event graphs, decomposing them into topologically connected subgraphs, and assigning alternative splicing event types (Table S1, available as supplementary data at *Bioinformatics* online). The final output provides detailed statistical measurements and event annotations.

SpliceHarmonization (Fig. 1) integrates splice detection outputs from rMATS, LeafCutter, and MAJIQ. As input, we extract specific elements from the output of each method and standardize them into a table format (Fig. 1A). For rMATS, each row corresponds to a predefined splicing event, from which we extract the event type, genomic coordinates of the involved exons, and associated statistical measurements. For LeafCutter and MAJIQ, each row represents an individual splice junction, defined by its donor and acceptor coordinates. Although these junctions are organized into splice clusters (LeafCutter) or local splicing variations (LSVs; MAJIQ), we retain only the junction-level information: donor and acceptor sites, corresponding cluster or LSV identifiers, and statistical metrics.

Based on these inputs, SpliceHarmonization framework proceeds through two key steps (Fig. 1B). First, rMATS splicing events are converted into a junction-centered representation to ensure compatibility with the junction-based outputs from LeafCutter and MAJIQ. Each junction is then mapped to both a reference transcriptomic annotation and a de novo genome annotation assembled by StringTie (Pertea *et al.* 2015). This alignment ensures consistency in genomic context across splicing detection methods and enables the integration of both annotated and novel splicing events within a unified coordinated framework (Britto-Borges *et al.* 2021). Second, using the collected junctions, SpliceHarmonization reconstructs splice graphs based on structural grouping information provided by each method. In rMATS, the splice graph is derived from predefined splicing event types; in LeafCutter, from splice cluster identifiers; and in MAJIQ, from LSV identifiers. From these method-specific splice graphs, SpliceHarmonization extracts a simplified subgraph, referred to as the event graph (Fig. 1B), by truncating each structure to a composition of three nodes and two edges.

This simplification approach is conceptually similar to the VOILA Modulizer (Vaquero-Garcia *et al.* 2023) but employs fewer event type annotations (event types shown in Fig. 1B; Table S1, available as supplementary data at *Bioinformatics* online). This simplified event graph is designed to capture the minimal local splicing contrast required to define a quantifiable alternative splicing event, while maintaining compatibility with event-centric outputs from widely used splicing tools. Each event can be unambiguously represented as a single local decision between two alternative splice paths (edges) with shared anchors (nodes), ensuring that it corresponds to change in transcript structure that can be robustly quantified using junction- or exon-level evidence across datasets.

The selection of event types in this work is motivated by the known mechanisms of small molecule splicing modulation. Small molecule modulators primarily alter pre-mRNA processing by perturbing splice-site recognition and spliceosome assembly, leading to discrete and local changes in exon or intron usage. In practice, such perturbations are most frequently presented as exon skipping or partial intron retention, resulting in altered composition of the mature RNA. Accordingly, exon inclusion/exclusion and intron retention provide direct and interpretable readouts for assessing both on-target and off-target splicing effects. In addition, modulation of splice-site recognition can give rise to alternative 5′ and 3′ splice-site usage, consistent with established bulge-repair and splice-site competition mechanisms at exon–intron boundaries. These events reflect localized shifts in splice-site selection. Based on these mechanistic considerations, this study focuses on four canonical splicing events: cassette exons (ESin and ESout), alternative 5′ splice sites (A5SSlong and A5SSshort), alternative 3′ splice sites (A3SSlong and A3SSshort), and intron retention (IRin and IRout), as summarized in Fig. 1B and Table S1, available as supplementary data at *Bioinformatics* online. More complex multi-junction or transcript-level splicing patterns, while detectable by MAJIQ, are not the primary targets of selectivity analysis in this context and are therefore not explicitly modeled as unified events. When a simplified subgraph (Section 1, available as supplementary data at *Bioinformatics* online) is paired with its corresponding event annotation (if available), it is labeled as an rMATS-Event, LeafCutter-Event, or MAJIQ-Event, and is hereafter referred to as an event module. SpliceHarmonization encompasses all events detected by any of these modules.

As a direct consequence of this junction-centered abstraction, certain exon boundary information is no longer explicitly represented. For example, when converting exon-centric quantification in rMATS to a junction-centric representation, the genomic boundaries of the leftmost and rightmost exons within a subgraph are not explicitly encoded. Similarly, LeafCutter and MAJIQ, by design, operate on splice junctions without reference to annotated exon boundaries. As a result, in our integrated method, each splicing event is recommended to be defined solely by junction coordinates (e.g. 1R-2L-2R-3L), with intron retention represented as a single junction (e.g. 1R-3L). These labeling conventions are illustrated in Fig. S1, available as supplementary data at *Bioinformatics* online. SpliceHarmonization delivers refined values for $\Psi_{\mathrm{ref}},\;\Psi_{\mathrm{alt}},\;\Delta \Psi$ and corresponding confidence scores, along with event types to describe alternative splicing across different methods (Fig. 1B). Here, Ψ (Percent Spliced In) represents the relative inclusion of a splice junction, ranging from 0 to 1. $\Psi_{\mathrm{ref}}$ and $\Psi_{\mathrm{alt}\;}$ denote the inclusion level in the control and treatment samples. ΔΨ, defined as $\Psi_{\mathrm{alt}}-\Psi_{\mathrm{ref}}$, describes the differential inclusion events between two sample groups. For rMATS and LeafCutter, the confidence score is calculated as 1 − FDR, and while for MAJIQ, it is equivalent to the posterior changing probability (P).

SpliceHarmonization does not simplify the splice graph for mutually exclusive exon (MXE) events; however, any adjacent pair of spliced-in and spliced-out exon events should theoretically be considered potential MXE events. This functionality has been implemented in a helper script. This pipeline is built for high-performance computing (HPC) environments and uses the power of bash scripting along with the analytical strengths of Python and R. The pipeline also employs parallel processing, enabling efficient analysis of splice events, which is important for identifying transcriptome-wide splice changes.

### 2.2 RNA-seq sample preparation for SpliceHarmonization framework

To prepare the inputs for SpliceHarmonization, upstream processing begins with quality control of raw RNA-seq data using FastQC, followed by read alignment to the reference genome using STAR (Dobin *et al.* 2013), which produces coordinate-sorted BAM files. These BAM files serve as the input for the SpliceHarmonization pipeline, which automates the entire downstream workflow and generates fully processed, harmonized splicing event outputs. As part of this workflow, splicing analysis is performed using rMATS, LeafCutter, and MAJIQ, whose outputs are internally integrated by SpliceHarmonization. In parallel, StringTie is used for de novo transcriptome assembly, supplemented by reference annotation GTF files to map transcripts accurately. Detailed parameters for these tools are available in Tables S1–S6, available as supplementary data at *Bioinformatics* online. RNA-seq data utilized in this study are downloaded from NCBI Sequence Read Archive (SRA), with accession number SRP334251(Ottesen *et al.* 2023).

### 2.3 Benchmarking SpliceHarmonization against widely used methods for splice events identification through simulation

Experimental validation on real datasets is limited to curated positive events and lacks genome-wide true negatives (TN), precluding unbiased quantitative benchmarking using real data alone. We therefore developed a splicing event simulator with a well-defined ground truth (Section 2, available as supplementary data at *Bioinformatics* online) to enable unbiased performance comparison across rMATS, LeafCutter, MAJIQ, and SpliceHarmonization. In the simulation, we randomly selected 128 genes to generate a diverse set of alternative splicing events (TrueEvent.csv, Supplementary Data, available as supplementary data at *Bioinformatics* online). Given that most splicing events influenced by splicing modulators involve exon skipping or inclusion, we focused specifically on exon inclusion (ESin) or exon exclusion (ESout). For each transcript, we simulated read counts at six levels (200, 500, 2000, 5000, 20 000, and 50 000 reads per transcript), under four alternative-to-control transcript ratios (100%:0%, 80%:20%, 50%:50%, and 20%:80%; see Fig. S2, available as *supplementary* data at Bioinformatics online). We further examine cross-method agreement using selected RT-PCR-validated splicing events (Ottesen *et al.* 2023).

Although SpliceHarmonization builds on the outputs of rMATS, LeafCutter, and MAJIQ, it performs additional harmonization steps, including coordinate realignment, junction-centric representation, and event graph construction, to enable consistent and interpretable downstream analyses. To evaluate the performance of SpliceHarmonization in comparison to the original methods, we considered the raw outputs (denoted as rMATS-Baseline, LeafCutter-Baseline, and MAJIQ-Baseline) and the structured event modules (rMATS-Event, LeafCutter-Event, and MAJIQ-event) produced by the SpliceHarmonization framework. Based on a ΔΨ cutoff, we defined true positives (TP) as correctly identified differential splicing events, false positives (FP) as non-changing events misclassified as changing, TN as correctly identified non-changing events, and, and false negatives (FN) as changing events misclassified as non-changing. Using these definitions, we computed the percentage of identified events, as well as accuracy, precision, recall, and under the receiver operating characteristic curve (AUC) for seven categories: rMATS-Event, rMATS-Baseline, LeafCutter-Event, LeafCutter-Baseline, MAJIQ-Event, MAJIQ-Baseline, and SpliceHarmonization.

Notably, rMATS provides event type annotations directly coupled with ΔΨ and FDR estimates, enabling straightforward evaluation of differential splicing events in the rMATS-Baseline. While MAJIQ also provides event type annotations associated with ΔΨ and posterior changing probability (P), for consistency with our simplified evaluation framework, we used the summary.tsvoutput from the VOILA Modulizer for the MAJIQ-Baseline analysis. This file includes only events that meet predefined thresholds for ΔΨ and P. In contrast, the raw output of LeafCutter does not include event type annotations. Consequently, for LeafCutter-Baseline analysis, we adopted a coarser event definition based on individual intron junctions matching, using the maximum absolute ΔΨ to represent splicing event identification. As a result, TN are undefined in both LeafCutter-Baseline and MAJIQ-Baseline analyses. In the LeafCutter-Baseline, events are defined by matching individual intron junctions to those in the simulated ground truth. Since the evaluation begins with the set of true event templates, and LeafCutter does not explicitly report non-changing junctions or provide event-level ΔΨ or FDR, it is not feasible to systematically identify TN; i.e. junctions correctly classified as non-changing. In the MAJIQ-Baseline, the use of the threshold-filtered summary.tsv output means that only positive calls are captured, without measures on confidently non-changing events. Thus, both methods lack the necessary outputs to identify TN, precluding the calculation of AUC for these two categories. This limitation underscores a critical challenge in the baseline methods, as the lack of TN can inflate performance metrics, thereby complicating direct comparisons with event modules. We note that the relative performance of splicing analysis methods depends on the dataset, splicing event type, evaluation metric, and that no single method consistently achieves optimal performance across all benchmarks.

## 3 Results

### 3.1 Inconsistent detection of differentially spliced genes across various methods necessitates a harmonized splice identification approach

**Figure 2:**
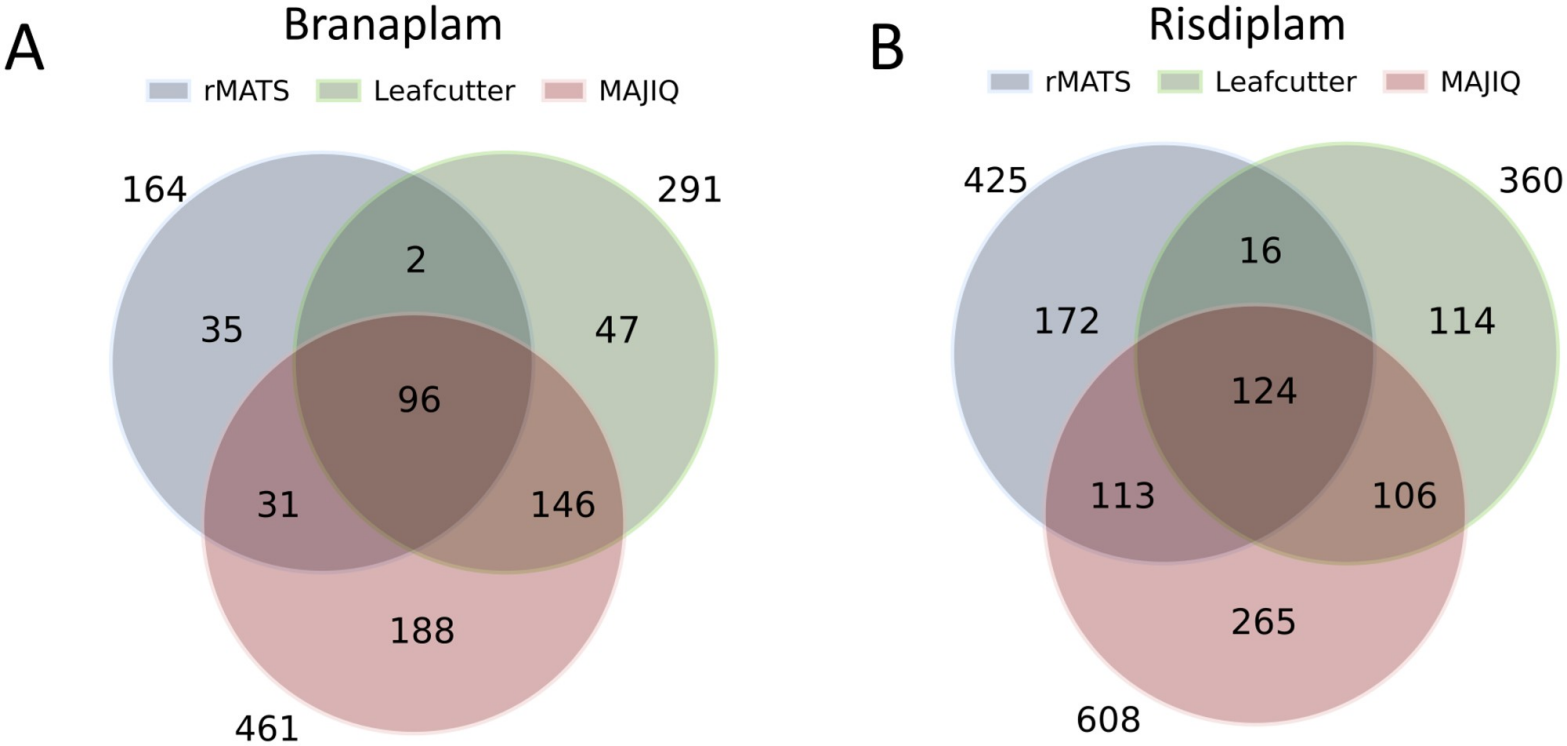
Variation in the detection of differentially spliced genes among splicing analysis methods. Venn diagram of identified differential splice genes from three different methods: rMATS, LeafCutter, and MAJIQ under (A) high-dose Branaplam (40 nM) and (B) high-dose Risdiplam (1000 nM).

We analysed a dataset generated from spinal muscular atrophy (SMA) patient-derived cells treated with SMN2-directed splicing modulators, Risdiplam and Branaplam (Ottesen *et al.* 2023). The original study investigates the effects of four treatment conditions: low (2 nM) and high (40 nM) doses of Branaplam versus DMSO, as well as low (50 nM) and high (1000 nM) doses of Risdiplam versus DMSO. In this work, we focused exclusively on the high-dose treatments: 40 nM Branaplam and 1000 nM Risdiplam. Using absolute ΔΨ > 0.2 and FDR < 0.05 cutoffs, we observed that the overlap of differentially spliced genes (DSGs) identified by each method was less than 50% of the total DSGs detected (Fig. 2). For example, the splice alteration in the HTT gene was not detected by rMATS in the high-dose Branaplam comparison, whereas both LeafCutter and MAJIQ identified modulation of this gene. To determine whether these discrepancies were inherent to the methods rather than due to differences in cutoff selection, we systematically evaluated various ΔΨ thresholds while maintaining an FDR < 0.05 for simplicity. Notably, even when evaluating all combination of method-specific ΔΨ cutoffs ranging from 0.1 to 0.9 (in increments of 0.1) for rMATS, LeafCutter, and MAJIQ, the best intersection over union (Jaccard index) across the three methods remained low, in which 19% for 40 nM Branaplam and 15% for 1000 nM Risdiplam in the context of a sufficiently large number of shared DSGs (Fig. S1; Tables S7 and S8, available as supplementary data at *Bioinformatics* online). Additionally, the combination of ΔΨ cutoff of 0.2 for all three methods are identified among the top 10 ranked ΔΨ threshold combinations that maximize the shared DSG set. These results emphasize that the limited overlap results from inherent differences in the splicing detection methods rather than the thresholds applied. Therefore, robust identification of compound-induced splicing changes is an important consideration during discovery of therapeutic splice modulators, as modulation of genes outside of the intended target, can pose safety concerns. Our harmonization method reduces Type II errors over using either one of the splice algorithms alone, allowing for comprehensive detection of DSGs without sacrificing specificity.

### 3.2 SpliceHarmonization provides a detailed and integrated analysis of differential splicing events

**Figure 3:**
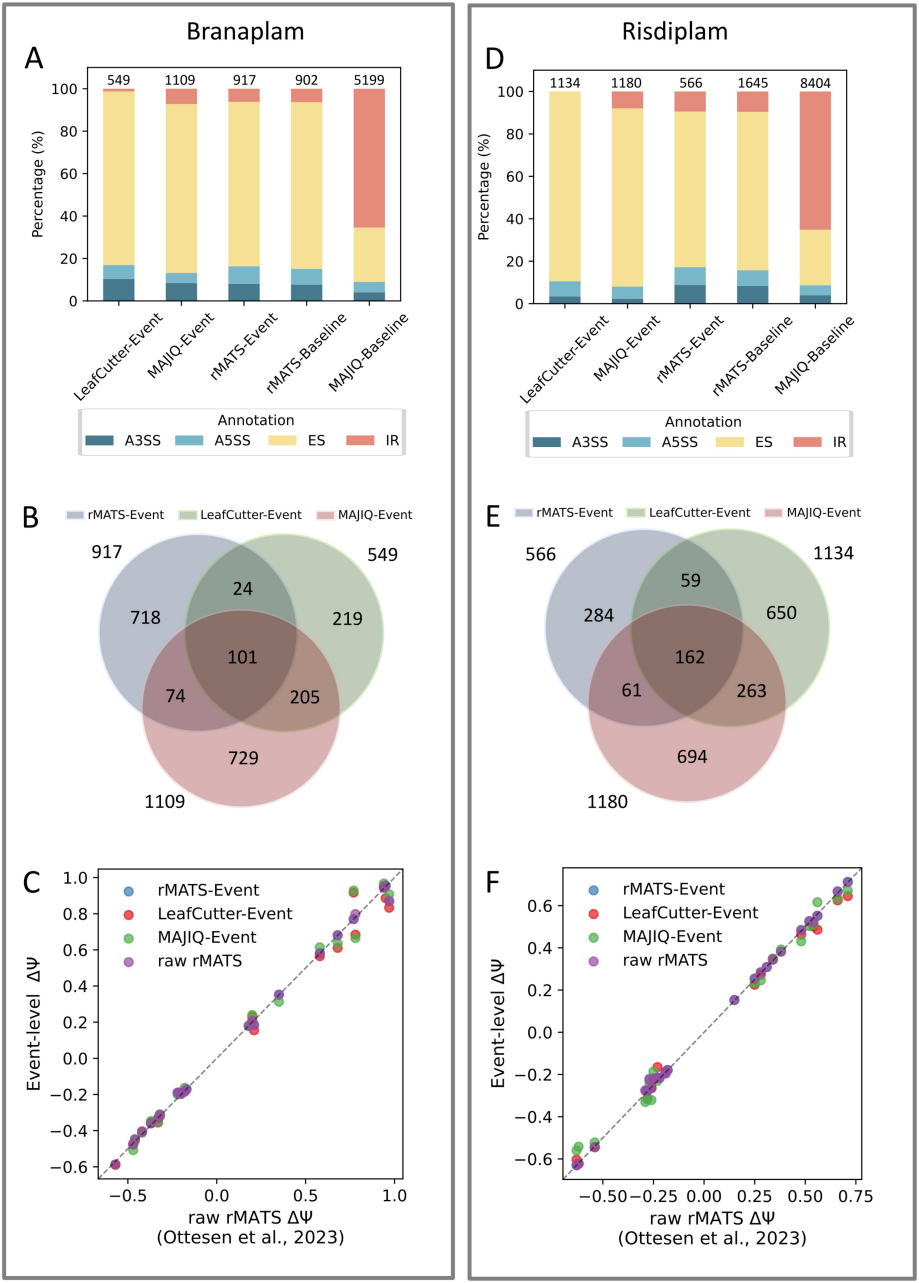
Event-level comparison of splicing detection across methods. (A and D) Distribution of splicing event annotations under treatment with 40 nM Branaplam (A) and 1000 nM Risdiplam (D), as detected by rMATS-Event, LeafCutter-Event, MAJIQ-Event, rMATS-Baseline, and MAJIQ-Baseline. Absolute event counts are labeled above each bar. (B and E) Venn diagram of identified splicing events from three different methods: rMATS-Event, LeafCutter-Event, and MAJIQ-Event under (B) high-dose Branaplam (40 nM) and (E) high-dose Risdiplam (1000 nM). (C and F) Cross-method comparison of event-level ΔΨ estimates for RT-PCR-validated splicing events with 40 nM Branaplam (C) and 1000 nM Risdiplam (F).

In Section 3.1, we highlighted discrepancies in identifying DSGs across methods. Here, we extend the analysis to examine how each method identifies and annotates specific splicing events, with a focus on discrepancies at the event level. To compare the distribution of annotated event types across methods, we analysed splicing changes under high-dose Branaplam and Risdiplam treatments using a cutoff of Δ*Ψ* > 0.2 and FDR < 0.05 (Fig. 3A and D). The comparison included five methods: rMATS-Event, LeafCutter-Event, MAJIQ-Event, rMATS-Baseline, and MAJIQ-Baseline. Because only original rMATS and MAJIQ provide event type annotations, original LeafCutter outputs splice clusters, making it difficult to directly profile individual splicing events. Among these, rMATS-Event, LeafCutter-Event, MAJIQ-Event, and rMATS-Baseline exhibited broadly similar event annotation distributions, as illustrated by the stacked percentage bar plots (Fig. 3A and D). In contrast, MAJIQ-Baseline showed a markedly different profile, with over 50% of its detected events classified as intron retention. Additionally, under both Branaplam and Risdiplam treatment conditions, the MAJIQ-Baseline consistently detected more splicing events than its event module.

To further evaluate the consistency in event detection across methods, we examined the overlap among three harmonized event modules: rMATS-Event, LeafCutter-Event, and MAJIQ-Event (Fig. 3B and E). This analysis revealed substantial discrepancies. For each method, at least 50% of the identified splicing events were not detected by the other two. This comparison was restricted to event modules, which obtain standardized and structurally comparable representations of splice events. In contrast, original outputs were excluded due to their inconsistent coordinate systems and method-specific definitions, which hinder direct comparison in the absence of a known ground truth. Overall, these findings underscore the need to develop an integrated method capable of precisely characterizing every potential splice event.

Because RT-PCR validation provides event-level confirmation of splicing changes induced by splicing modulators (Ottesen *et al.* 2023) but does not define genome-wide quantitative ground truth, analyses on this experimental dataset focus on directional concordance and cross-method consistency rather than benchmarking (Tables S9 and S10, available as supplementary data at *Bioinformatics* online). Since ΔΨ values from LeafCutter-Baseline and MAJIQ-Baseline describe the junction-level splicing changes, they are not directly comparable to RT-PCR measurements, while ΔΨ values from rMATS-Baseline, by definition, represent event-level splicing changes. Accordingly, only rMATS-Baseline and structured event modules (rMATS-Event, LeafCutter-Event, and MAJIQ-Event) were included in this analysis. Both Fig. 3C and F demonstrate that SpliceHarmonization enables unified event-level comparison across heterogeneous splicing detection methods on experimentally validated events.

### 3.3 The integrated SpliceHarmonization method outperforms single splice identification methods in identified events, recall, and AUROC

To evaluate the integrated event-level analysis, we developed a splicing event simulator for comparing multiple RNA-seq experiments (Section 2, available as supplementary data at *Bioinformatics* online). In these simulations, we introduced 128 alternative splice events randomly selected (Table 1). Among these, 23 events contained cryptic exons, while 105 events were annotated as defined in the reference GTF. Most of the simulated splice events were modeled as exon inclusion (ESin) or exon exclusion (ESout), reflecting the nature of splice modulators.

**Table 1:** Simulated splice event distribution.

Splice event annotation	Number of events
Exon inclusion (ESin)	69
Exon exclusion (ESout)	28
5′ splice-site elongation (A5SSlong)	10
5′ splice-site shrinkage (A5SSshort)	5
3′ splice-site elongation (A3SSlong)	2
3′ splice-site shrinkage (A3SSshort)	4
Intron retention spliced in (IRin)	6
Intron retention spliced out (IRout)	4

**Figure 4:**
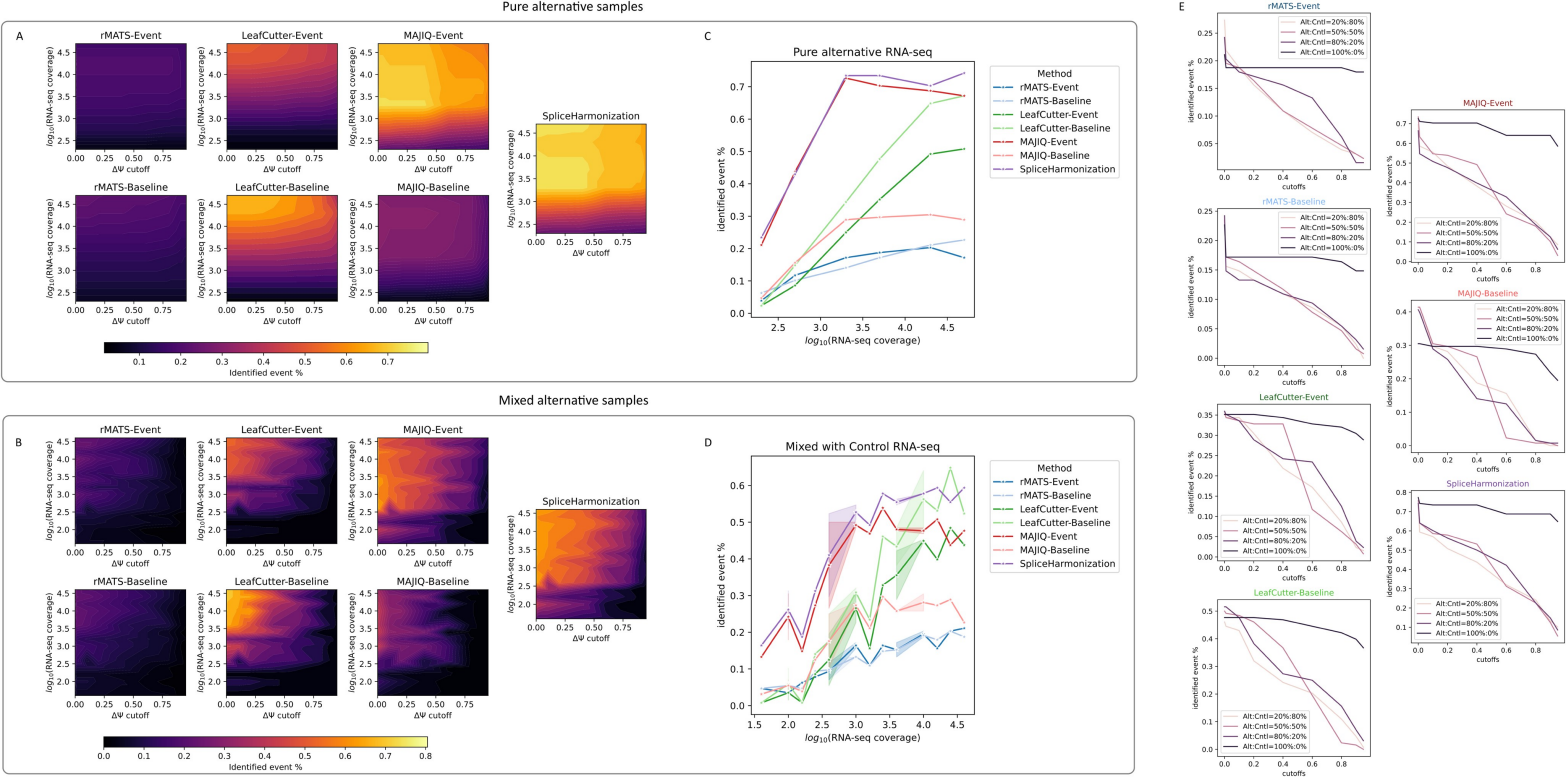
Splice identification performance across simulated RNA-seq alternative coverages and ΔΨ thresholds. Panels (A and B) show the identification percentage (TP events divided by 128 true events) as a function of $\log_{10}($RNA-seq coverage) (*y*-axis) and ΔΨ (*x*-axis). Panels (C and D) demonstrate the splice identification under different $\log_{10}($RNA-seq coverages) when ΔΨ = 0.2. Data in (A and C) are from pure alternative samples (alternative: control ratio = 100%:0%), whereas (B and D) show results for mixed alternative samples (ratios of 80%:20%, 50%:50%, and 20%:80%). Panel (E) shows the variation in the identified splicing event % as a function of cutoff thresholds across different alternative:control ratios and methods.

For the purposes of classifier evaluation, we simplified the analysis by exclusively relying on the ΔΨ threshold and disregarding the FDR value as a cutoff criterion. Using the specified ΔΨ, we classified events into TP, FP, TN, and FN across rMATS-Event, rMATS-Baseline, LeafCutter-Event, LeafCutter-Baseline, MAJIQ-Event, MAJIQ-Baseline, and SpliceHarmonization methods. Figure 4A and B illustrates the splice identification percentage (defined as TP divided by the total number of true events, *n* = 128) across various simulated RNA-seq coverage (*y*-axis) and ΔΨ (*x*-axis). Specifically, data from pure alternative samples (alternative: control ratio = 100%:0%) are displayed in Fig. 4A, while results from mixed alternative samples with ratios of 80%:20%, 50%:50%, and 20%:80%. RNA-seq alternative coverage was calculated by multiplying the number of reads per transcript by the percentage of the alternative transcript in Fig. 4B; for instance, an alternative-to-control ratio of 80%:20% with 5000 reads yields a coverage of 5000 × 0.8 = 4000. In pure alternative samples (alternative:control = 100%:0%), the percentage of detected splicing events remains constant across varying ΔΨ, as shown by the plateau observed in Fig. 4E. This indicates that strong splicing signals are relatively insensitive to changes in ΔΨ. Conversely, for mixed alternative samples, increasing the ΔΨ cutoff leads to a reduction in the percentage of detected events, demonstrating the weak splicing signals are more sensitive to the stringency of the threshold (Fig. 4E, alternative:control = 80%:20%, 50%:50%, and 20%:80%). Consequently, we adopted a ΔΨ of 0.2 for subsequent analyses. In summary, SpliceHarmonization consistently detected the highest number of events under varying coverage and ΔΨ.

When a ΔΨ threshold of 0.2 was applied (Fig. 4C and D), SpliceHarmonization achieved the highest percentage of splice event identification, followed by MAJIQ and LeafCutter, with rMATS trailing. Moreover, the comparable performance observed between rMATS-Baseline and rMATS-Event indicates that the event graph preserves the integrity of the original rMATS output. In contrast, LeafCutter-Event detected fewer events than LeafCutter-Baseline, while MAJIQ-Event detected more events than MAJIQ-Baseline. This discrepancy results from three factors: first, the coarse nature of the event identification in the baseline method; second, subgraph simplification in event-based methods averages scores that will shift the ΔΨ and FDR, compared to the original methods; and third, shifts of several base pairs relative to the genomic annotation, which further exacerbate the discrepancy. Additionally, at low read coverage levels, MAJIQ detected a number of events comparable to those identified by LeafCutter and rMATS; however, as the read coverage increased, LeafCutter’s detection rate improved markedly, while rMATS lagged behind.

**Figure 5:**
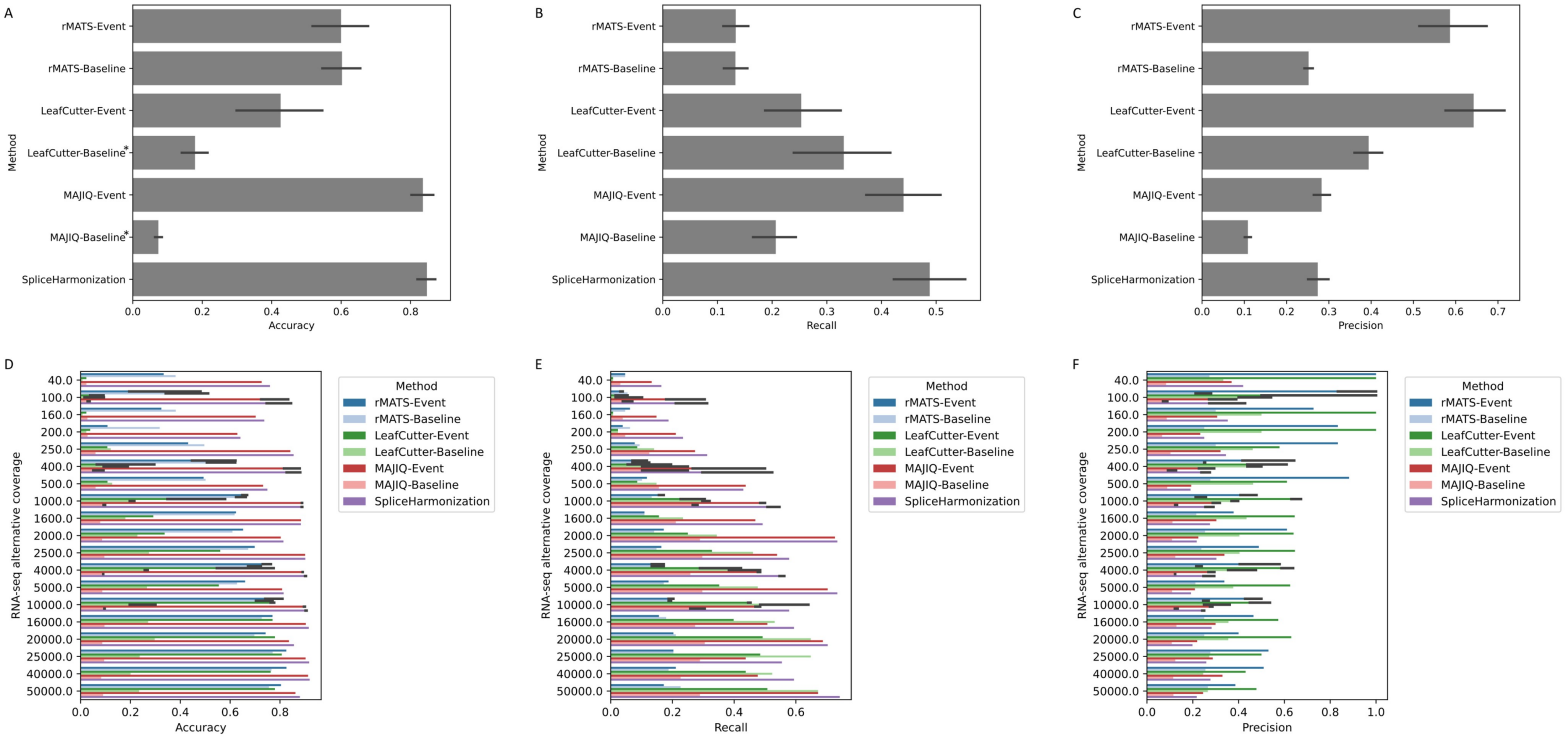
Benchmarking metrics for method comparison. Panels (A–C) display the mean values of accuracy, recall, and precision, respectively, for the various methods under the condition of ΔΨ = 0.2. In panel (A), asterisks denote that the measurements for LeafCutter-Baseline and MAJIQ-Baseline are unreliable due to a true negative count of zero. Panels (D–F) illustrate the variation in these metrics across different levels of RNA-seq alternative coverage for the same methods at ΔΨ = 0.2.

The accuracy, recall, and precision at ΔΨ = 0.2 are recapitulated in Fig. 5. Considering accuracy (Fig. 5A and D), the outputs of SpliceHarmonization and MAJIQ-Event were nearly identical, with a slight improvement observed at high coverage levels (i.e. >20 000 reads). At very low coverage (<250 reads), LeafCutter-Event was unable to detect splice events unlike the other methods. However, at coverage levels exceeding 10 000 reads, LeafCutter-Event achieved accuracy comparable to that of SpliceHarmonization, MAJIQ-Event and rMATS-Event. Additionally, as mentioned in Section 2.3, the absence of true negative event identification in both LeafCutter-Baseline and MAJIQ-Baseline substantially reduces their accuracy; therefore, these two values should be interpreted with caution and are excluded from comparative evaluation. For recall (Fig. 5B and E), SpliceHarmonization achieved the highest score due to its integration of outputs from all three methods, followed sequentially by MAJIQ-Event and LeafCutter-Event. Specifically, the recall performance of rMATS-Event was comparable to that of the rMATS-Baseline, as was the case for LeafCutter-Event relative to its baseline. In contrast, MAJIQ-Event exhibited a higher recall than MAJIQ-Baseline, corresponding to a greater number of detected true events relative to the simulated ground truth. The event graph approach, as illustrated in Fig. 5C and F, also affected precision. While it tended to reduce false positive counts across all event-based approaches, this reduction enhanced overall precision when compared to the baseline methods (Fig. S2, available as supplementary data at *Bioinformatics* online). Moreover, because our integration approach increased both FP and TP simultaneously, the net effect was a decrease in precision.

**Figure 6:**
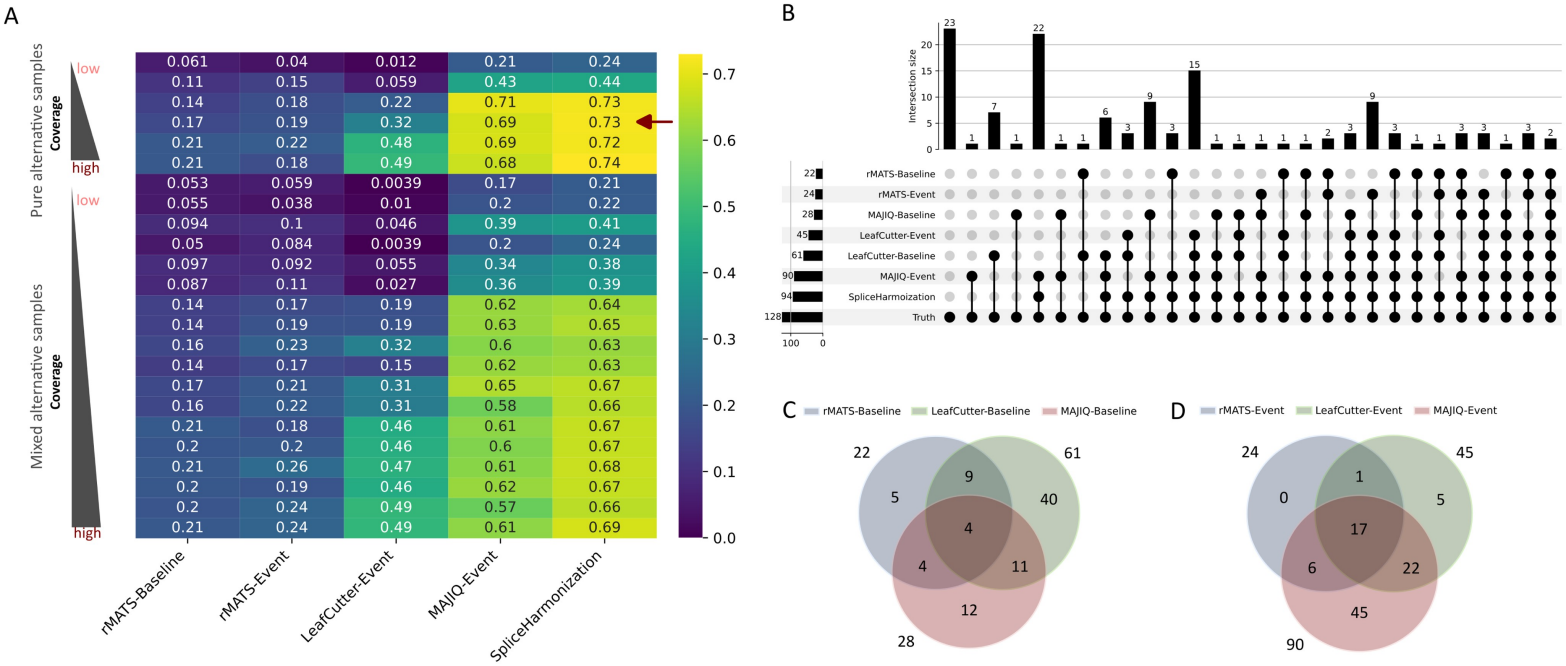
Method performance and overlap in detecting differential splicing events. (A) Heatmap illustrating the area under the ROC curve (AUROC) values across various read coverage and event detection methods. The AUCROC is computed as the integral of the true positive rate against the false positive rate across a series of ΔΨ threshold (0, 0.01, 0.1, 0.2, 0.4, 0.6, 0.8, 0.9, 0.95) under the conditions of varying simulated reads per transcript (200, 500, 2000, 5000, 20 000, and 50 000) derived from pure alternative samples (alternative:control ratio = 100%:0%) or mixed alternative samples (ratios of 80%:20%, 50%:50%, and 20%:80%). The area under the curve (AUCROC) quantifies the overall discriminative ability of different methods shown in heatmap with higher value means the better performance. (B–D) Overlap analysis of true positive splicing event detection by different methods from pure alternative samples with 5000 simulated reads per transcript and ΔΨ > 0.2 (indicated by the arrow in panel (A)). (B) Upset plots quantifying the intersection across eight sets, thereby delineating method-specific and shared events. (C and D) Venn diagram illustrating the pairwise overlaps among splicing events detected by rMATS, LeafCutter, and MAJIQ using two distinct approaches. Panel (C) presents the pairwise overlaps among events identified through the baseline approach, whereas panel (D) illustrates the overlaps when a simplified graph-based approach is employed. This consolidated view enables a direct comparison of the impact of methodological variations on the shared detection of high-confidence events with ΔΨ > 0.2.

Collectively, these findings suggest that SpliceHarmonization offers the most robust overall performance, followed by standalone usage of MAJIQ, LeafCutter, and rMATS. This trend is further corroborated by the AUROC heatmap presented in Fig. 6A. Because AUROC calculations also rely on positive and negative classes, AUROC values for Leafcutter-Baseline and MAJIQ-Baseline are inherently constrained by the absence of true negative events (Section 2.3) and should therefore be interpreted with caution. Interestingly, our workflow significantly enhanced splicing event prediction for MAJIQ, as demonstrated by the improved outcomes observed with MAJIQ-Event compared to the MAJIQ-Baseline (Figs 4–6). Meanwhile, LeafCutter-Baseline showed good performance at high read coverage (>10 000 reads per transcript), but its event annotation remains relatively coarse. Moreover, after applying the simplified graph-based approach, the number of true positive events commonly detected by any two or all three methods increased significantly compared to those observed with the original approaches (Fig. 6C and D). This finding indicates the SpliceHarmonization enhances the concordance between different methods, potentially leading to more robust predictions of true splicing events.

## 4 Conclusions and discussion

Here, we present SpliceHarmonization, an integrated splicing event identification method that benchmarks the output of rMATS, LeafCutter and MAJIQ. Through benchmarking against individual methods, we demonstrated that SpliceHarmonization outperforms other methods by providing a comprehensive event analysis and highest identified events percentage, recall values, and AUROC scores. Unlike LeafCutter, which provides junction-level statistics, SpliceHarmonization assigns event type annotations to junctions identified from rMATS, LeafCutter, and/or MAJIQ through harmonized event modules. Importantly, this framework is extensible and can be applied to additional splice analysis methods, regardless of whether they are event-centric like rMATS or junction-centric like LeafCutter and MAJIQ.

Existing methods for integrating results from multiple splicing tools differ substantially in their scope and level of integration. Meta-analysis approaches typically aggregate tool-specific outputs in a post hoc manner without reconciling differences in event definitions or splicing representations and are therefore challenged by heterogeneous coordinate systems across exon-centric and junction-centric methods. Frameworks such as Baltica (Britto-Borges et al. 2021) provide standardized workflows and unified junction coordinates for comparing outputs from multiple splicing tools but focus primarily on junction-level summaries and do not explicitly harmonize splicing events. In contrast, SpliceHarmonization introduces a unified event graph representation that enables structural integration of splicing events, supporting direct event-level comparison across tools for assessing the effects of splicing modulators.

One limitation of SpliceHarmonization is its reliance on accurate genomic annotation to unify the junction coordinates, making it sensitive to any base pair shifts. In applications such as splicing modulators screening, where sensitivity is critical and safety is of concern, it is essential that the harmonization method achieves the highest recall. Conversely, in context where minimizing FP is a priority, LeafCutter-Event may be preferable due to its higher precision. It is also worth noting that SpliceHarmonization focuses on major event types such as exon skipping/inclusion, retained intron inclusion/skipping, and alternative 5′/3′ splice-site usage, while the original MAJIQ method remains better suited for detecting more complex splicing patterns, including tandem cassette exons and multi-exon spanning events. Furthermore, in this study, the MAJIQ-Baseline analysis was based on its summary-level splicing event output. For a more accurate and comprehensive comparison, future evaluations may benefit from incorporating MAJIQ’s full output, which includes detailed statistical measurements for all detected splicing events.

## Supplementary Material

btag111_Supplementary_Data

## Data Availability

SpliceHarmonization is available at https://github.com/interactivereport/SpliceHarmonization.
