# Supplementary material for: SpliceHarmonization: an integrated method for identifying RNA splicing events in therapeutics for splicing modulation
Source: Bioinformatics. 2026 Mar 20;42(4):btag111. doi: 10.1093/bioinformatics/btag111 (PMC13064980; doi:10.1093/bioinformatics/btag111)
Supplement: btag111_Supplementary_Data [file btag111_supplementary_data.zip › FINAL_Supplemenary Materials.docx]

Supplementary Materials

Table S1: STAR alignment

| Parameters | |
| --- | --- |
| Reference | Human.GRCh38.v34.l1_5.ERCC |
| alignIntroMin | 20 |
| alignIntronMax | 1000000 |
| alignMatesGapMax | 1000000 |
| alignSJoverhangMin | 8 |
| alignSJDBoverhangMin | 1 |
| outFilterMismatchNmax | 999 |
| outFilterMismatchNoverLmax | 0.05 |
| outFilterMultimapNmax | 20 |
| outFilterType | BySJout |
| outSAMunmapped | Within |

Table S2: RSEM

| Parameters |  |
| --- | --- |
| estimate-rspd | true |
| quiet | false |
| fragment-length-mean | 150 |
| fragment-length-sd | 35 |
| strandedness | reverse |

Table S3: rMATS

| Parameters |  |
| --- | --- |
| t | paired |
| readLength | 100 |
| Ramts_variable_read_length | false |
| cstat | 0.0001 |
| rmats_paired_stats | false |
| rmats_novel_splice_sites | false (SMN2); true (simulation) |
| rmats_allow_clipping | false |

Table S4: Leafcutter

| Parameters |  |
| --- | --- |
| leafcutter_anchor_len | 8 |
| leafcutter_min_intron_len | 50 |
| leafcutter_max_intron_len | 50000 |
| leafcutter_min_cluster_reads | 30 |
| leafcutter_min_samples_per_intron | 2 |
| leafcutter_min_samples_per_group | 2 |
| leafcutter_plots_fdr | 0.05 |
| leafcutter_max_plots | 30 |

Table S5: Majiq

| Parameters | defaults |
| --- | --- |

Table S6: Stringtie

| Parameters |  |
| --- | --- |
| stringtie_min_cov | 3 |
| stringtie_min_jxn_cov | 3 |
| stringtie_min_isoform_frac | 0.001 |

Table S7. Top 10 ranked threshold combination maximizing the similarity among spliced genes identified by three methods (shared count > 80) in 40 nM Branaplam.

| ΔΨ(rMATS) | ΔΨ(LeafCutter) | ΔΨ(MAJIQ) | Jaccard Index  rMATS:LeafCutter | Jaccard Index  rMATS:MAJIQ | Jaccard Index  LeafCutter:MAJIQ | Shared gene count | Union gene count | Jaccard Index  (three methods) |
| --- | --- | --- | --- | --- | --- | --- | --- | --- |
| 0.2 | 0.2 | 0.3 | 0.27 | 0.28 | 0.50 | 86 | 458 | 0.19 |
| **0.2** | **0.2** | **0.2** | **0.27** | **0.26** | **0.47** | **96** | **545** | **0.18** |
| 0.2 | 0.2 | 0.1 | 0.27 | 0.24 | 0.46 | 96 | 575 | 0.17 |
| 0.1 | 0.1 | 0.1 | 0.28 | 0.23 | 0.45 | 157 | 984 | 0.16 |
| 0.2 | 0.1 | 0.2 | 0.21 | 0.26 | 0.43 | 114 | 729 | 0.16 |
| 0.2 | 0.1 | 0.1 | 0.21 | 0.24 | 0.45 | 115 | 747 | 0.15 |
| 0.1 | 0.1 | 0.2 | 0.28 | 0.22 | 0.43 | 149 | 971 | 0.15 |
| 0.2 | 0.1 | 0.3 | 0.21 | 0.28 | 0.37 | 98 | 662 | 0.15 |
| 0.1 | 0.2 | 0.1 | 0.19 | 0.23 | 0.46 | 126 | 882 | 0.14 |
| 0.1 | 0.2 | 0.2 | 0.19 | 0.22 | 0.47 | 120 | 858 | 0.14 |

Table S8. Top 10 ranked threshold combination maximizing the similarity among spliced genes identified by three methods (shared count > 80) in 1000 nM Risdiplam.

| ΔΨ(rMATS) | ΔΨ(LeafCutter) | ΔΨ(MAJIQ) | Jaccard Index  rMATS:LeafCutter | Jaccard Index  rMATS:MAJIQ | Jaccard Index  LeafCutter:MAJIQ | Shared gene count | Union gene count | Jaccard Index  (three methods) |
| --- | --- | --- | --- | --- | --- | --- | --- | --- |
| 0.1 | 0.1 | 0.1 | 0.29 | 0.25 | 0.32 | 285 | 1870 | 0.15 |
| 0.2 | 0.1 | 0.1 | 0.21 | 0.30 | 0.32 | 207 | 1390 | 0.15 |
| 0.2 | 0.1 | 0.2 | 0.21 | 0.30 | 0.30 | 196 | 1360 | 0.14 |
| 0.1 | 0.1 | 0.2 | 0.29 | 0.22 | 0.30 | 255 | 1852 | 0.14 |
| **0.2** | **0.2** | **0.2** | **0.22** | **0.30** | **0.31** | **124** | **910** | **0.14** |
| 0.2 | 0.2 | 0.1 | 0.22 | 0.30 | 0.30 | 128 | 957 | 0.13 |
| 0.1 | 0.2 | 0.1 | 0.14 | 0.25 | 0.30 | 159 | 1591 | 0.10 |
| 0.1 | 0.2 | 0.2 | 0.14 | 0.22 | 0.31 | 148 | 1568 | 0.09 |
| 0.2 | 0.1 | 0.3 | 0.21 | 0.21 | 0.15 | 99 | 1264 | 0.08 |
| 0.3 | 0.1 | 0.1 | 0.09 | 0.16 | 0.32 | 93 | 1285 | 0.07 |

Table S9. Event-level splicing changes ($\Delta\Psi$) detected by multiple methods under 40 nM Branaplam treatment with RT-PCR validation

Please see the separate csv file: TableS9.HiB_validation_summary.csv

Table S10. Event-level splicing changes ($\Delta\Psi$) detected by multiple methods under 1000 nM Risdiplam treatment with RT-PCR validation

Please see the separate csv file: TableS10.HiR_validation_summary.csv

| 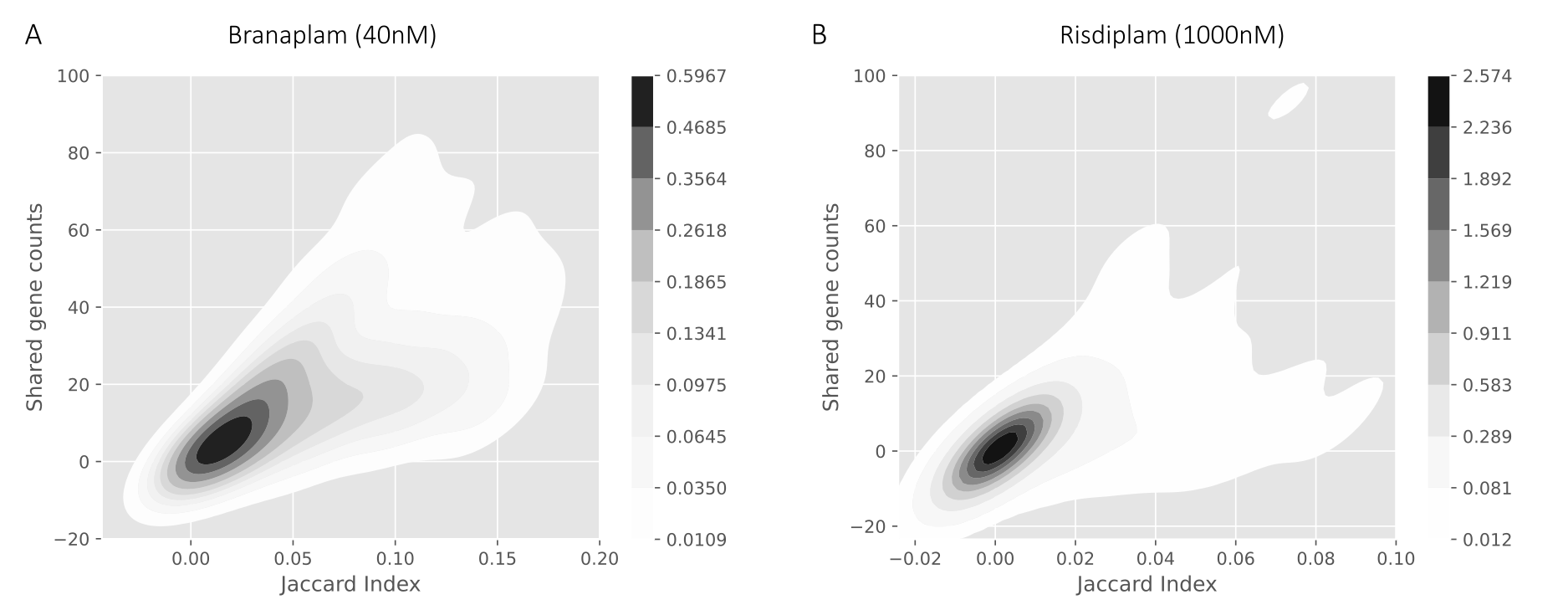 |
| --- |
| Figure S1. Kernel density estimation (KDE) plot illustrating the joint distribution of the Jaccard Index, defined as the ratio of the shared spliced gene count to the union spliced gene count derived from three methods (rMATS, LeafCutter, MAJIQ), and the shared gene count for splicing events or junctions that meet various $\Delta\Psi$ threshold combinations and FDR < 0.05 in (A) 40 nM Branaplam and (B) 1000 nM ridisplam |

|  |
| --- |
| 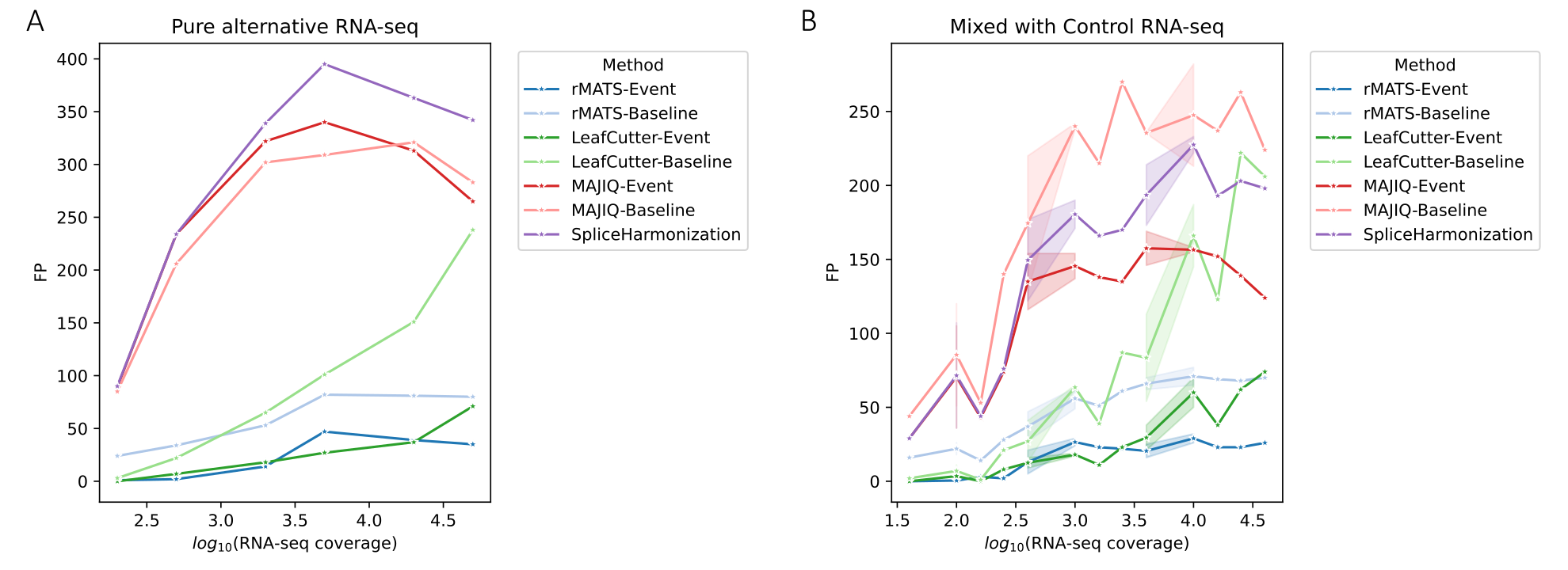 |
| Figure S2. False positive count profiles for various splicing detection methods at different $\log_{10} (RNAseq)$ when ΔΨ= 0.2 from (A) pure alternative samples (alternative:control ratio = 100%:0%); (B) mixed alternative samples (ratios of 80%:20%, 50%:50%, and 20%:80%). |
