# Supplementary material for: SpliceHarmonization: an integrated method for identifying RNA splicing events in therapeutics for splicing modulation
Source: Bioinformatics. 2026 Mar 20;42(4):btag111. doi: 10.1093/bioinformatics/btag111 (PMC13064980; doi:10.1093/bioinformatics/btag111)
Supplement: btag111_Supplementary_Data [file btag111_supplementary_data.zip › FINAL_Supplementary Methods.docx]

Supplementary Methods

1. **Graph Simplification**

ConnectedTriplets(G)

| Algorithm ConnectedTriplets (𝐺) |
| --- |
| Input:  Graph 𝐺 = (𝑉, 𝐸) represented as an adjacency list |
| Output:  List of all connected triplets in 𝐺  Initialized connected Triplets ← ∅  for each unique combination (u,v,w) of three distinct nodes in 𝑉:  If IsConnectedSubgraph(u,v,w) = ture then  connectedTriplets ← connectedTriplets ∪ {(𝑢, 𝑣, 𝑤)} |

| Function IsConnectedSubgraph (𝑢, 𝑣, 𝑤, 𝐺) |
| --- |
| Input:  Nodes 𝑢, 𝑣, 𝑤 and Graph 𝐺 = (𝑉, 𝐸) |
| Output:  Boolean value indicating whether {𝑢, 𝑣, 𝑤} forms a connected subgraph  Initialize visited ← ∅  Initialize 𝑄 ← {𝑢}  Mark 𝑢 as visited (visited ← visited ∪ {𝑢}.)  while 𝑄 ≠ ∅:  𝑥 ← dequeue(𝑄)  for each neighbor 𝑛 in neighbors(𝑥):  if (𝑛 ∈ {𝑢, 𝑣, 𝑤}) and (𝑛 ∉ visited):  Mark 𝑛 as visited (visited ← visited ∪ {𝑛})  Enqueue 𝑛 into 𝑄  if visited = {𝑢, 𝑣, 𝑤}:  return True  else:  return False |

Table S1: Splice event annotations in SpliceHarmonization

| Event Types in Figure 1B | Splice Event Annotation |
| --- | --- |
| ESin | Exon inclusion |
| ESout | Exon exclusion |
| A5SSlong | 5’ splice site elongation |
| A5SSshort | 5’ splice site shrinkage |
| A3SSlong | 3’ splice site elongation |
| A3SSshort | 3’ splice site shrinkage |
| IRin | Intron retention spliced in (IRin) |
| IRout | Intron retention spliced out (IRout) |

1. **Splice Event Simulation Workflow**
   1. Input specifications
      1. Input spreadsheet

Required inputs include the gene name, chromosomal location, strand orientation, six coordinates (Cassette 1L, Cassette 1R, Cassette 2L, Cassette 2R, Cassette 3L and Cassette 3R), and the event annotation (example please see TrueEvent.csv).

- - 1. Six coordinate rules

The six coordinates correspond to distinct splice events, as outlined in Figure S1.

- 1. RNAseq simulation
     1. Extraction of FASTA Files

FASTA were extracted separately from the reference FASTA file for both the alternative and control samples.

- - 1. Generation of Simulated RNA-seq Data

Simulated RNA-seq FASTA data were generated using the Polyester (Frazee, et al., 2015), with the parameters detailed in Table S2.

- - 1. Conversion to FASTQ Format

The simulated FASTA file was converted into a FASTQ file using 'I' for Phred score 40

- - 1. Sequence Alignment

The FASTQ sequences were aligned using the STAR aligner to produce BAM files, employing the parameters specified in Table S3.

- 1. Outputs

The process yields BAM files along with curated event metadata (see TrueEvent.csv).

In this study, we simulated read counts at six levels: 200, 500, 2000, 5000, 20,000, and 50,000 reads per transcript, across four alternative-to-control transcript ratios (100:0%, 80:20%, 50:50%, and 20:80%). Figure S2 illustrate the effect of different alternative-to-control transcript ratios on simulations.

Table S2: Polyester

| Control simulation | |
| --- | --- |
| num_reps | 3 |
| reads_per_transcript | [200, 500, 2000, 5000, 20000, 50000] |
| fold_changes | 3 |
| Alternativesimulation | |
| num_reps | 3 |
| reads_per_transcript | $c(\left( 1-r_{a2c} \right)*P_{\mathrm{ref}}, r_{a2c}*P_{\mathrm{alt}})$  where P samples from [200, 500, 2000, 5000, 20000, 50000],  $r_{a2c}$ represents alternative:control ratio varies from 0.2, 0.5, 0.8 and 1. |
| fold_changes | 3 |

Table S3: STAR alignment

| Parameters |  |
| --- | --- |
| runMode | alignReads |
| reads_per_transcript | 20 |
| outSAMtype | BAM Unsorted |
| outReadsUnmapped | Fastx |

|  |
| --- |
| Figure S1. Six genomic coordinates (cassette1L, cassette1R, cassette2L, cassette2R, cassette3L, cassette3R) are depicted, each corresponding to an independent splice event. These coordinates provide the formatted input for splice event simulations. |

|  |
| --- |
| Figure S2. Splice simulation of the MYO1B gene displaying a skipped exon highlighted by a red box. Simulated read counts of 5000 per transcript were visualized in IGV. The first track represents the control sample, while the second to fifth tracks correspond to alternative-to-control transcript ratios of 20%:80%, 50%:50%, 80%:20%, and 100%:0%, respectively. Variations in these ratios yield differential coverage of the skipped exon. |

Reference

Frazee, A.C.*, et al.* Polyester: simulating RNA-seq datasets with differential transcript expression. *Bioinformatics* 2015;31(17):2778-2784.
